# A new species of *Zachaeus* C.L. Koch from Turkey (Opiliones, Phalangiidae)

**DOI:** 10.3897/zookeys.514.9545

**Published:** 2015-07-22

**Authors:** Kemal Kurt, Halil Koç, Ersen Aydın Yağmur

**Affiliations:** 1 Gümüşhane University, Şiran Vocational High School, TR-29700 Gümüşhane, Turkey; 2 Sinop University, Science and Art Faculty, Biology Department, Sinop, Turkey; 3 Alaşehir Vocational School, Celal Bayar University, TR-45600, Alaşehir, Manisa, Turkey

**Keywords:** Opiliones, Phalangiidae, *Zachaeus*, new species, Turkey

## Abstract

A new species of harvestmen, *Zachaeus seyyari* **sp. n.** (Opiliones, Phalangiidae), is described and illustrated on the basis of both sexes from Şırnak Province in Turkey. Differences between the new species and related species are indicated. Photographs of its characteristic structures are also provided.

## Introduction

*Zachaeus* C.L. Koch, 1839 is a genus belonging to the subfamily Phalangiinae of the Phalangiidae and it is distributed in the eastern part of the Mediterranean Region, south-eastern Europe, and western Asia (Snegovaya and Staręga 2008). The genus includes 12 species: *Zachaeus anatolicus* (Kulczynski, 1903), *Zachaeus birulai* (Redikorzev, 1936), *Zachaeus crista* (Brullé, 1832), *Zachaeus hebraicus* (Simon, 1884), *Zachaeus hyrcanus* (Redikorzev, 1936), *Zachaeus kervillei* (Sørensen, 1912) species inquirenda, *Zachaeus lupatus* (Eichwald, 1830), *Zachaeus mirabilis* (Caporiacco, 1949), *Zachaeus orchimonti* (Giltay, 1933), *Zachaeus redikorzevi* (Staręga & Chevrizov, 1978), *Zachaeus shachdag* (Snegovaya & Staręga, 2008), and *Zachaeus simferopolensis* (Chemeris & Kovblyuk, 2005) [Giltay 1933; Redikorzev 1936; Caporiacco 1949; Snegovaya and Staręga 2008], of which five are known in Turkey: *Zachaeus anatolicus*, *Zachaeus crista*, *Zachaeus hebraicus*, *Zachaeus orchimonti*, and *Zachaeus redikorzevi* (Kurt, 2014).

The genus is characterized by the following morphological characteristics: body large, heavily denticulated dorsally; chelicerae usually strong, second segment enlarged; pedipalps normally structured, strong, robust; legs short and first pair much thicker than the others; truncus penis basally widened, parallel-sided on the distal half, distally shallow spoon-shaped, glans usually banana-shaped (shorter in *Zachaeus anatolicus* and *Zachaeus seyyari*), stylus long (Snegovaya and Staręga 2008, 2009). Here, we describe a new species of the genus *Zachaeus* from Şırnak Province in Turkey, and compare it to the most similar species.

## Material and methods

Samples were collected by hand from the meadows and grassland in Şırnak Province, Turkey. Species identification was conducted using a Leica EZ4 stereomicroscope. Specimens are preserved in 70% ethanol and deposited in the collection of the Arachnological Laboratory of Şiran Vocational School, Gümüşhane University (GUSAL), Turkey. All measurements are given in millimeters.

## Results

### Taxonomy Family Phalangiidae Latreille, 1802 Genus *Zachaeus* C.L. Koch, 1839

#### Zachaeus seyyari sp. n.

Taxon classificationAnimaliaOpilionesPhalangiidae

http://zoobank.org/89BE1BA3-8AEE-4CB1-A1F2-17B789F8BB6C

Figs 1 , 2 , 3 , 4

##### Type material.

**Holotype**: 1♂ (GUSAL), Turkey: Şırnak Province, İdil District, Yörük Village (37°16'47.54"N, 42°1'17.18"E), 655 m, 12 May 2007, leg. E.A. Yağmur and H. Koç.

**Paratypes.** 2 ♂, 4♀ (GUSAL), 1 ♂, 1♀ (AZMM=Alaşehir Zoological Museum, Manisa) same data as holotype.

##### Distribution.

Up to now only known from type locality in the Şırnak Province, Turkey.

##### Diagnosis.

The new species is similar to *Zachaeus anatolicus* (Kulczyński 1903: 660; Šilhavý 1956: 34, figs 1–5; -1965: 382–384, figs 1–13, Staręga 1976: 376, figs 75–77; Chevrizov 1979: 22, figs 119–121) and *Zachaeus redikorzevi* (Staręga and Chevrizov 1978: 419–422, figs 1–2; Staręga 1978: 219; Chevrizov 1979: 22, figs 122–124; Kurt et al. 2011: 146–147, figs 1–8). The differences between these species are given in Table 1.

**Table 1.** Main diagnostic characters of most closely related species in the genus *Zachaeus*.

Characters	*Zachaeus seyyari* sp. n.	*Zachaeus anatolicus*	*Zachaeus redikorzevi*
Body	cephalothorax dorsally with small denticles; abdomen dorsally not denticulated	cephalothorax and abdomen dorsally with numerous denticles (Staręga 1976).	cephalothorax dorsally only granulated on surface, abdomen dorsally not denticulated (Staręga and Chevrizov 1978)
Tuber oculorum	relatively low and 1–2 setae in two rows	low and 4–8 tubercles in two rows (Staręga 1976).	low and 5–6 tubercles in two rows (Staręga and Chevrizov 1978)
Chelicerae of male	second segment swollen (more cylindrical), covered with setae.	second segment not swollen, covered with setae and microdenticles (Staręga 1976).	second segment extraordinarily swollen, covered with setae and microdenticles (Staręga and Chevrizov 1978)
Palp of male	patella dorsally with setae; tibia with setae.	patella dorsally with microdenticles, tibia with setae and microdenticles (Staręga 1976).	patella dorsally with setae; tibia only with setae (Staręga and Chevrizov 1978)
Leg	femur I–III with setae; femur IV ventrally with denticles, dorsally setae.	femur I–IV with denticles (Staręga 1976).	femur I–III with setae; femur IV ventrally with denticles (Staręga and Chevrizov 1978)
Penis	truncus wide at the base, basal to center with straight sides; then slightly narrowed at the center; then not widened, straight-sided at the subapex; glans stocky, not elongated, parallel sided, ventrally slightly oval-sided, apical outline rectangular.	truncus wide at the base, base to center narrowed; then widened at the subapex; glans, stocky, not elongated, ventrally oval (Staręga 1976).	truncus slightly enlarged at the proximal half; then straight-sided in distal half; glans elongated and narrow, ventrally oval-sided, apical outline triangular (Staręga and Chevrizov 1978)

##### Derivatio nominis.

The specific epithet is in honor of Dr. Osman SEYYAR (Niğde University, Niğde, Turkey), who has made important contributions to Turkish arachnology.

##### Description.

Male: body length 7.2 mm, width 4.5 mm; chelicera basal segment 2.8 mm, second segment 3.7 mm.

**Figure 1.**
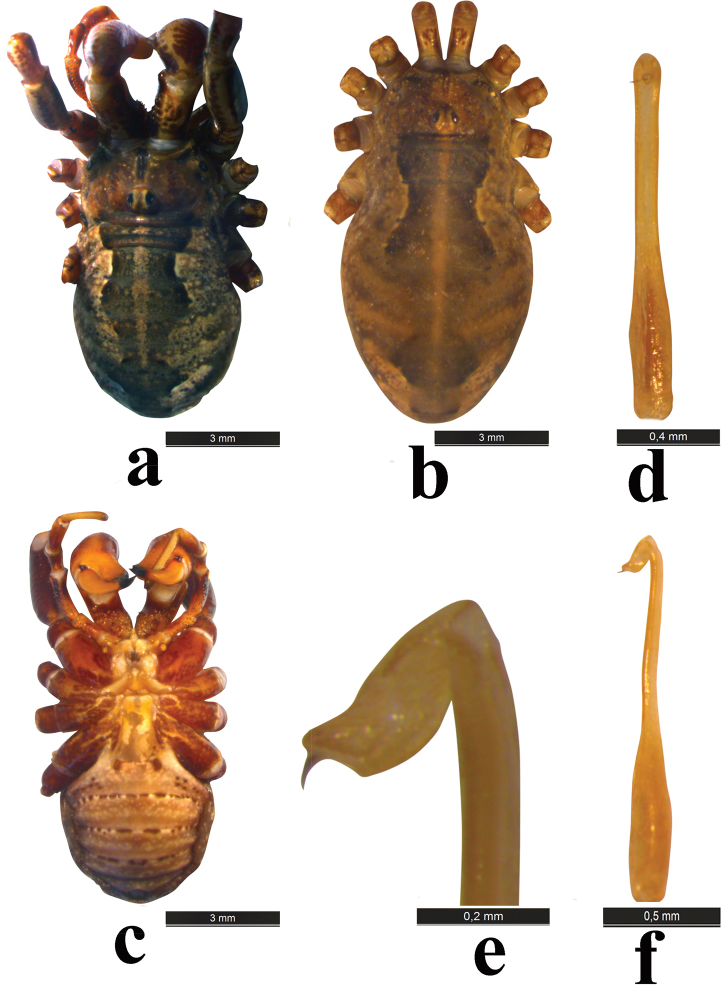
*Zachaeus seyyari* sp. n.: **a** body, male, dorsal view **b** body, female, dorsal view **c** body, male, ventral view **d** penis, dorsal view **e** glans, lateral view **f** penis, lateral view.

Body (Fig. 1a): approximately oval-shaped in dorsal view. Opening of odoriferous gland prominent with 1–2 black denticles. Cephalothorax covered with small black denticles. Carapace ochre-brown. Abdomen dorsally with distinct brownish-gray saddle. Saddle with longitudinal whitish-yellow stripe in the center. Abdominal tergites with transverse rows of dark brown spots, not denticulated.

Tuber oculorum (Fig. 4f): nearly hemispherical, median furrow present, relatively low and with 1–2 setae on each side.

Ventral side (Fig. 1c): coxae and genital operculum covered with sparse hairs. Abdomen ventrally with transverse rows of brown spots, and with sparse hairs.

**Figure 2.**
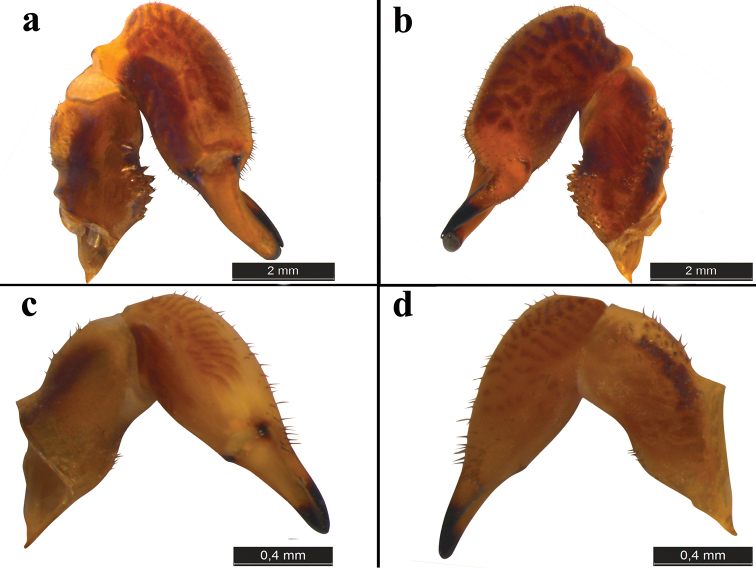
Chelicerae of *Zachaeus seyyari* sp. n.: **a, b** chelicerae, male, lateral view **c, d** chelicera, female, lateral view.

Chelicerae (Fig. 2, 4e): strong, robust and dark ochre brown. Basal segment apically not widened and slightly bent, dorsally with small black-tipped tubercles and setae, ventrally with long black-tipped tubercles. Second segment apically widened, zebra-like stripe pattern of pigmentation, and covered with setae.

**Figure 3.**
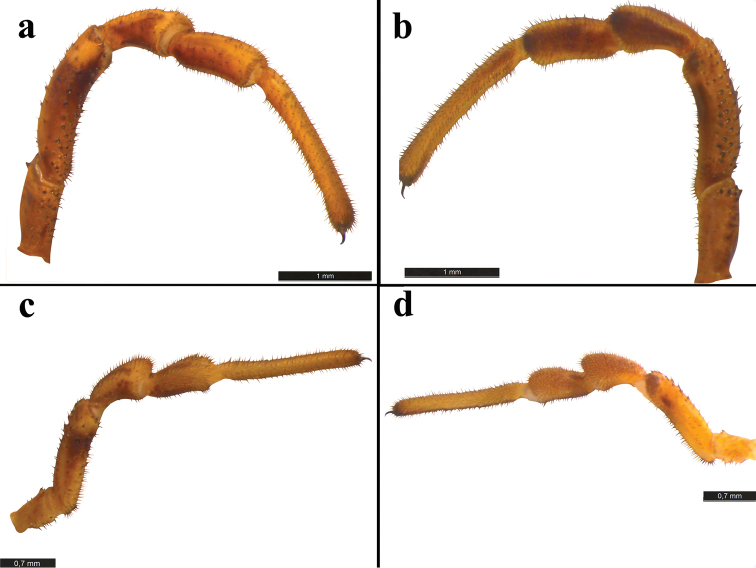
Pedipalp of *Zachaeus seyyari* sp. n.: **a, b** pedipalp, male, lateral view **c, d** pedipalp, female, lateral view.

Pedipalp (Fig. 3): normally structured, strong; ochre-brown and with dark brown spots. Coxae with finger-shaped apophysis and covered with long setae; trochanter relatively long, ventrally and dorsally with black tubercles and setae; femur of male slighty curved, dorsally and ventrally covered with black-tipped tubercles and setae; patella distally with usual bulge densely hairy in female, less developed in male: similar in tibia; tibia and tarsus only with setae, but male tarsus ventrally bearing black microdenticles, tarsal claw smooth.

**Figure 4.**
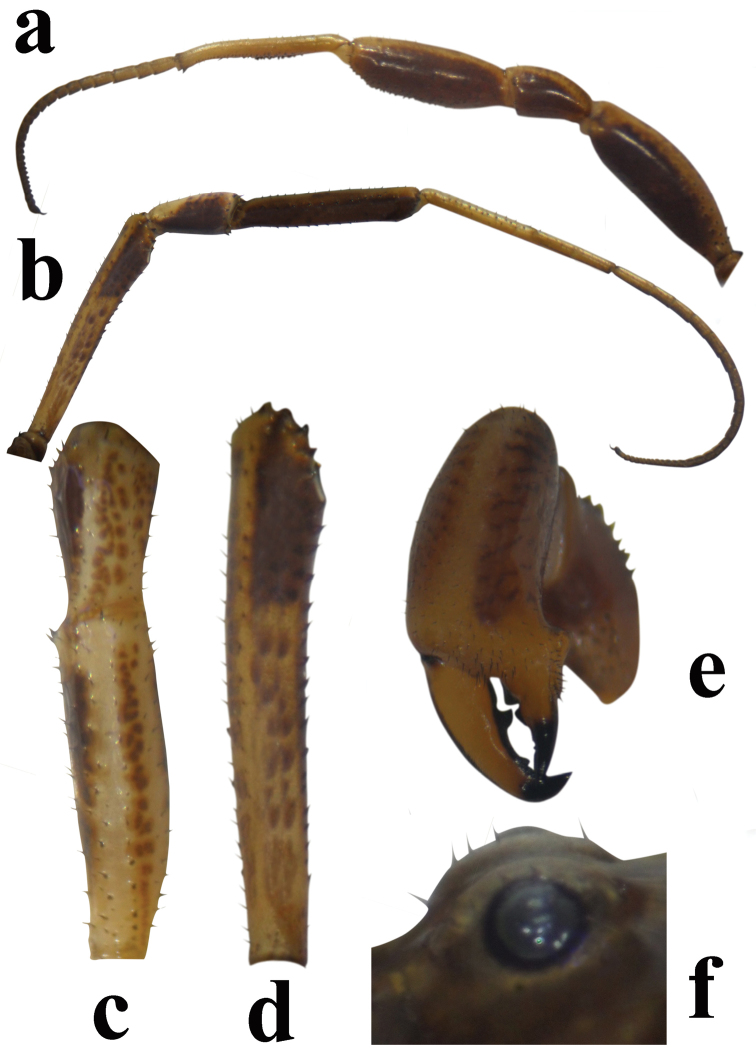
**a, b** Leg of *Zachaeus seyyari* sp. n.: **a** pair I, male, lateral view **b** pair IV, male, lateral view **c** pair I femur and patella, female, dorsal view **d** pair IV femur, lateral view **e** chelicerae, male, frontal view **f** tuber oculorum, male, lateral view.

Legs (Fig. 4a–d): short and strong, light ochre-brown and with dark brown spots. Femur to tarsus I relatively thicker than in legs II to IV. Femur and patella I with setae, tibia ventrally with microdenticles and dorsally with setae, metatarsus ventrally covered with densely spaced microdenticles, tarsus bearing only setae. Leg pairs II and III with sparse setae. Femur and tibia IV ventrally covered with black denticles, and dorsally setae; metatarsus IV ventrally with bristle, dorsally with microdenticles; tarsus ventrally bristle, dorsally with setae.

Male genital morphology (Fig. 1d–f): truncus wide at base, proximal fourth of shaft straight-sided; then slightly narrowed at the center; straight-sided from center to distal end of shaft, forming a spoon-shape, wings not very wide; glans stocky, widened, not elongated, not banana-shaped; stylus long.

Female: body length 9.0 mm, width 4.7 mm; chelicera basal segment 1.5 mm, second segment 2.1 mm. General appearance similar to that of male, but body larger and wider (Fig. 1b). Second segment of chelicerae normally structured, not enlarged, basal segment ventrally without tubercles.

**Table 2.** Measurements (in mm) of male holotype (female paratype).

	Femur	Patella	Tibia	Metatarsus	Tarsus	Total
Palp	1.7 (1.5)	0.9 (0.85)	1.2 (0.95)	- (-)	1.9 (1.7)	5.7 (5.0)
Leg I	3.2 (2.8)	1.4 (1.2)	2.94 (2.2)	2.7 (2.1)	4.2 (4.0)	14.44 (12.3)
Leg II	3.8 (3.4)	1.5 (1.4)	3.5 (3.1)	3.7 (3.1)	7.9 (7.8)	20.4 (18.8)
Leg III	2.3 (2.1)	1.3 (1.1)	2.5 (2.1)	3.0 (2.9)	4.8 (4.7)	13.9 (12.9)
Leg IV	3.5 (3.5)	1.4 (1.3)	3.3 (3.2)	3.5 (3.5)	7.2 (7.0)	18.9 (18.5)

##### Discussion.

This paper describes a new species belonging to the genus *Zachaeus*. This genus has five species (*Zachaeus anatolicus*, *Zachaeus crista*, *Zachaeus hebraicus*, *Zachaeus orchimonti*, and *Zachaeus redikorzevi*) in Turkey. *Zachaeus anatolicus* is distributed in Bulgaria, Caucasus, Crimea, Greece, former Yugoslavia, and Turkey (Adana, Ankara, Bayburt, Gümüşhane, Kayseri and Manisa Provinces) and *Zachaeus redikorzevi* is recorded from Russia and Turkey (Bayburt, Gümüşhane, and Osmaniye Provinces) (Kurt 2014). *Zachaeus crista* is distributed throughout South and Eastern Europe, Caucasus. It is widespread in Turkey (Ankara, Antalya, Bayburt, Bilecik, Bolu, Denizli, Gümüşhane, İzmir, Kırıkkale, Niğde and Osmaniye Provinces). *Zachaeus hebraicus* is known from Jordan, Israel, Lebanon, Libya, Syria and Turkey (Adana, Manisa Provinces). *Zachaeus orchimonti* is only known from Turkey (Aydın, Denizli, İzmir and Manisa Provinces) (Giltay, 1932) and *Zachaeus kervillei* is known from Syria (Roewer 1923). The new species differs from *Zachaeus hebraicus*, *Zachaeus crista*, and *Zachaeus kervillei* by the presence of setae only on the ocularium, abdomen not denticulated dorsally, and legs I–III femora covered with setae only (*Zachaeus hebraicus*, *Zachaeus crista*, and *Zachaeus kervillei* are characterized by ocularium with denticles, abdomen dorsally denticulated, and leg I–III femora covered with denticles). *Zachaeus seyyari* sp. nov. differs from *Zachaeus orchimonti* by a setose ocularium, femur of pedipalp dorsally and ventrally covered with black-tipped tubercles and setae (in *Zachaeus orchimonti*, ocularium with 5–7 small denticles and femur of pedipalp with setae).

With *Zachaeus seyyari* sp. n., the number of *Zachaeus* species known from Turkey is now increased to 6. Considering the geographical features of Turkeyand the habitat preferences of the genus, the number of species will surely increase with ongoing studies in the future.

## Supplementary Material

XML Treatment for Zachaeus seyyari
